# The Pocket Case of Drugs

**Published:** 1877-06-20

**Authors:** 


### THE POCKET CASE OF DRUGS.

We are aware that the country physician seldom carries a pocket case of drugs, using only the saddle bags. In this they impose upon themselves much inconvenience. The pocket case, if judiciously arranged, will enable the physician in a great degree, to dispense with the saddle bags As a sign or advertisement it may be well for the young physician to carry his saddle bags at all times when riding, but he will seldom have occasion to open them, if he carries a well-arranged pocket case. The pocket case furnishes a good index by which to judge of the qualifications of the practitioner. If he is of the old fogy list, and grown somewhat careless, his pocket case, if he has one, contains but three or four phials upon which are no labels; it is half worn out, stained and dirty, and his whole armamentarinm for combatting the multiform maladies of his fellowmen consists usually of a little *laudanum*, *quinine*, *Dover's powder* and *blue mass* or *calomel*. But if he reads the journals, and is a progressive man with a conscience alive to the duties and responsibilities of his profession, he will endeavor to avail himself of the late improvement, and the many new and valuable therepentical agents that have come to light, so that when a patient trusts his health and life to his skill and judgment he will be able to feel, whatever may be the result of the case, that he gave him all the advantages of the present state of medical science so far as he could obtain them, and his conscience will be void of offense: Otherwise he has trifled with human life, betrayed the confidence of his patient and dishonored himself and his calling.

But as the physician cannot carry a drug shop with him, he should select as far as possible, a list of the leading and most valuable articles, in a form compact, neat, ready and convenient. While it is admitted that the selection in regard to remedies of the same class, may vary with the views of different men, the one for instance selecting as an astringent tannin, and another kino, etc., yet there ought to be in the general outline, an approximate uniformity, which, in variety, would give expression to the present status of therepeutics as held by the leading and progressive minds in the profession. Without extending our remarks on this subject we subjoin our view of a pocket case, and what it should contain:

Let the case consist of a double row of ten, two-drachm, phials, making twenty in all, and a lapel pocket in it, for papers and powders; the phials to be filled and labeled as below—the saddle-bags to contain the same in larger quantities, with cups, an elastic syringe, a pocket-case of instruments, adhesive plaster, a little patent lint, blistering ointment, or fluid, and, in the vest pocket, a hypodermic syringe:

POCKET-CASE OUTFIT.

| | |
|---|---|
| Tinc. Aconite, | Hydrag. Cum. Creta, |
| Tinc. Gelseminum, | Creta Preparata, |
| Tinc. Veratrum, | Podophyllin, |
| Deod. Tinc. Opii, | Pulv. Doveri, |
| Tinc. Wild Yam, | Acetate of Lead, |
| Carbolic Acid, | Sulph. Quinine, |
| Nit Silver, | Chloroform, |
| Fluid Ext. Ergot, | Hydrate of Chloral, |
| Ipecac, | Brom. Potassium, |
| Sacharated Calomel, | Sulph. Morphine. |

A little tannic acid, subnitrate of bismuth, and santonine may be carried, in the lapel-pocket of the case.

With this in your side-pocket, whether at church, at a picnic, or elsewhere, you are pretty well prepared for emergencies.

